# Risk factors for unplanned and unwanted teenage pregnancies occurring over two years of follow-up among a cohort of young South African women

**DOI:** 10.3402/gha.v7.23719

**Published:** 2014-08-21

**Authors:** Nicola J. Christofides, Rachel K. Jewkes, Kristin L. Dunkle, Frances McCarty, Nwabisa Jama Shai, Mzikazi Nduna, Claire Sterk

**Affiliations:** 1 School of Public Health, Faculty of Health Sciences, University of the Witwatersrand, Johannesburg, South Africa; 2 Gender and Health Research Unit, Medical Research Council, Pretoria, South Africa; 3 Rollins School of Public Health, Behahioral Sciences and Health Education, Emory University, Atlanta, GA, USA; 4 Department of Psychology, University of the Witwatersrand, Johannesburg, South Africa

**Keywords:** unplanned pregnancy, unwanted pregnancy, adolescent pregnancy, gender-based violence, South Africa

## Abstract

**Background:**

Although teenage pregnancies in South Africa have declined, the short and longer term health and social consequences are a potential public health concern. This longitudinal study aimed to describe the range of risk and protective factors for incident unwanted and unplanned pregnancies occurring over 2 years of follow-up among a cohort of adolescent women in the Eastern Cape, South Africa. It also investigated the relationship between gender inequality and gender-based violence and subsequent unplanned and unwanted pregnancies among the cohort.

**Objective:**

Teenage girls, aged 15–18 years (*n*=19), who were volunteer participants in a cluster randomized controlled trial and who had data from at least one follow-up were included in this analysis. To assess risk and protective factors for incident unwanted or unplanned pregnancies, we constructed multivariate polytomous regression models adjusting for sampling clusters as latent variables. Covariates included age, having a pregnancy prior to baseline, education, time between interviews, study intervention arm, contraceptive use, experience of intimate partner violence, belief that the teenage girl and her boyfriend are mutual main partners, and socioeconomic status.

**Results:**

Overall, 174 pregnancies occurred over the 2-year follow-up period. Beliefs about relationship control were not associated with unwanted and unplanned pregnancies, nor were experiences of forced first sex or coerced sex under the age of 15. Hormonal contraception was protective against unplanned pregnancies (OR 0.40; 95% CI 0.21–0.79); however, using condoms was not protective. Physical abuse (OR 1.69; 95% CI 1.05–2.72) was a risk factor for, and having a pregnancy prior to baseline was protective against an unwanted pregnancy (OR 0.25; 95% CI 0.07–0.80). Higher socioeconomic status was protective for both unplanned and unwanted pregnancies (OR 0.69; 95% CI 0.58–0.83 and OR 0.78; 95% CI 0.64–0.96). Believing that the teenage girl and her boyfriend were mutual main partners doubled the odds of reporting both an unplanned and unwanted pregnancy (OR 2.58 95% CI 1.07–6.25, and OR 2.21 95% CI 1.13–4.29).

**Conclusion:**

Although some of the measures of gender inequity were not associated with unplanned and unwanted pregnancies, there is evidence of the role of both gender power and socioeconomic status. This was evident in teenage girls who experienced physical violence being more likely to have an unwanted pregnancy. Interventions to prevent teenage pregnancies need to be tailored by socioeconomic status because some teenagers may see having a pregnancy as a way to have a more secure future. Interventions that engage with relationship dynamics of teenagers are essential if unwanted and unplanned pregnancies are to be prevented.

Teenage pregnancies have adverse short- and long-term health outcomes for both the young women and their infants. Teenage mothers have been found to be at increased risk for anemia, urinary tract infection, and pregnancy-induced hypertension (1–4) . Infants were more likely to suffer infant and neonatal death, accidents, infections, and sudden infant death syndrome (SIDS) (5). There is also a body of literature that has explored the longer term social and mental health consequences of teenage pregnancy. These consequences include depression and substance use, increased sexual risk behavior, as well as lower educational attainment and socioeconomic status (6–8) . Most of this literature emanates from developed countries in North America, Australia, and Europe.

Nearly one-third of South African women report having had a teenage pregnancy (9, 10). There is evidence that teenage fertility declined by 10% between 1996 and 2001 from 79 births per 1,000 women to 65 births per 1,000 women (11).

Studies from South Africa have described the relationship between teen pregnancies and poorer educational outcomes (12, 13). A recent study of young women in South Africa found that early teenage pregnancies increased risk of acquiring HIV (14).

It is against this backdrop of both short and longer term adverse health outcomes that teenage pregnancies are largely viewed as a public health issue. However, few studies investigating the risk factors for teenage pregnancies differentiate between desired, unwanted, and unplanned pregnancies. In the South African context, some teenage pregnancies are desired, but most are unplanned or unwanted (11, 15). An unplanned pregnancy is described in the literature as a pregnancy that is not desired at that particular time, in other words it is mistimed, whereas an unwanted pregnancy is not wanted at all (16). The adverse consequences for unwanted pregnancies tend to be more severe than those for unplanned pregnancies (17). Literature that differentiates between unplanned and unwanted pregnancies has focused on women of all age groups and not on teenage girls in particular. Differentiating between unplanned and unwanted pregnancies may allow for a more nuanced understanding of the risk factors of teenage pregnancy, and may allow for the development of more closely targeted and more effective prevention strategies. In addition, increasing understanding of the difference between unplanned and unwanted pregnancy may assist health care providers in determining not only the unmet need for contraceptive services but also for abortion services.

Studies from South Africa have identified lower educational achievement and a shock to the household, defined as death of a household member, job loss, marital disruption, or loss of a grant or remittance, as a risk factors for teenagers becoming pregnant (12, 13). Other studies have found an association between gender inequality and gender-based violence, including child sexual abuse and forced first sex, and teenage pregnancies (15, 18–24) . However, this research has rarely differentiated on the bases of whether the teenage pregnancy was unplanned or unwanted.

This longitudinal study aimed to describe the range of risk and protective factors for incident unwanted and unplanned pregnancies occurring among a cohort of 819 teenage women aged 15–18 over approximately 2 years of follow-up. It also aimed to describe the relationship between gender inequality and gender-based violence and subsequent incident pregnancies among a cohort of teenage girls in the Eastern Cape, South Africa.

## Methods

### Study population and setting

In total, 1,416 South African women aged 15–26 were recruited from 70 villages and residential areas near Mthatha in the Eastern Cape, South Africa. The analysis presented here is based on a subset of 922 teenage women aged 15–18 years at baseline.

### Study design

Data for the analysis was from a cluster randomized controlled trial of the Stepping Stones HIV prevention intervention (25, 26). The study was a stratified, two-stage survey with villages sampled from predefined strata based on geographical characteristics and participants clustered within villages. Villages were eligible for inclusion if they were in the study area, located 10 km or more from other study villages, contained a secondary school, and were willing to participate. Once village gatekeepers agreed to inclusion, volunteers were recruited, primarily from secondary schools. Twenty women were recruited per cluster. All participants were resident in the village where they were schooling and mature enough to understand the content of the intervention (25). For the purposes of these analyses, the women were treated as an observational cohort, and exposure to the Stepping Stones intervention was treated as a covariate.

### Questionnaire

Detailed discussion of the development and content of the questionnaire appears elsewhere (25). Data used here include sociodemographic characteristics, sexual behavior, and a range of reproductive outcomes including pregnancy. Women who responded in the affirmative to the question ‘Since the previous interview have you been pregnant?’ or to the question ‘Are you currently pregnant?’ at either follow-up interview were categorized as having an incident pregnancy. An additional question asked when the respondent became pregnant. To eliminate any misclassification of the timing of pregnancies, the reported dates when a woman became pregnant and her baseline interview dates were compared to ensure that the pregnancy started after the baseline interview. Assessment of pregnancy desire was based on the response to the item: ‘At the time you became pregnant did you want to become pregnant then, did you want to wait until later, or did you not want to have any children at all?’ Women who responded that they ‘wanted to wait until later’ were classified as having an unplanned pregnancy whereas those who said that they ‘did not want to have children at all’ were classified as having an unwanted pregnancy. Teenage girls who said that they wanted the pregnancy at that time were dropped from further analysis (*n*=10). Many of the teenage girls had a pregnancy prior to the baseline assessment. For the analysis of the association between child sexual abuse and first pregnancies, all first pregnancies were included in the analysis, including those prior to baseline and those that occurred over the 2 years of follow-up.

The questionnaire included the WHO violence against women instrument which was modified to be culturally appropriate (27). The instrument included five items measuring single and multiple occurrences of physical abuse occurring within the past 12 months and over a woman's lifetime, and four items measuring single and multiple occurrences of sexual abuse within the past 12 months and over a woman's lifetime. Three variables measuring intimate partner violence (IPV) were derived. These included a 3-level categorical variable of type of abuse which included no abuse, physical only, and sexual abuse with or without physical abuse. The frequency of IPV was measured through a categorical variable with no abuse, once only, and more than once. The temporality of IPV was measured using a 4-level variable: no abuse, abuse occurring before the previous 12 months only, abuse occurring in the past 12 months only, and abuse occurring both before and during the past 12 months.

Coerced first sex was derived from an item that asked ‘Which of the following statements most closely describes your experiences the first time you had sexual intercourse? ‘I was willing; I was persuaded; I was tricked; I was forced; I was raped’. This was dichotomized into those who were willing and those who were coerced.

Child sexual abuse was measured through a response to one or more of four items that asked about the period ‘before the age of 18,’ for example ‘Someone touched my thighs, buttocks, breasts, or genitals when I did not want him to or made me touch his private parts when I did not want to’ (28).

For women with current main male partners at baseline, a sexual relationship power scale developed by Pulerwitz and her colleagues (10-items, Cronbach's alpha =0.73), previously shown to be associated with prevalent HIV among South African women, was used to measure gender power equity (29). A typical item was, ‘When (Name of boyfriend) wants me to sleep over, he expects me to agree’. Each item was assessed on a 4-point Likert scale and the measure was scored and categorized into tertiles. For the analyses, the tertile with lowest equity was compared to the middle and higher ones.

Contraceptive use was measured by an item that asked ‘Are you currently doing something or using any method to delay or avoid getting pregnant?’ and follow-up questions which asked about which contraceptive method was being used. Contraceptive use was then categorized into a 3-level variable: no contraceptive use, hormonal contraceptives, or condoms only. Contraceptive knowledge was measured using a 6-item scale, with items such as: ‘A woman who is not using contraception and has sex during her period will probably get pregnant’. The continuous variable was later dichotomized into higher and lower knowledge.

Three questions established past year numbers of main boyfriends, *khwapheni* (hidden partners concurrent with main partners), and men with whom the participant had sex only once. Socioeconomic status was assessed by use of a scale that encompassed household goods ownership, food, and cash scarcity.

### Data collection

Face-to-face interviews by trained, female interviewers using standardized questionnaires were carried out at 12-month intervals over approximately 2 years of follow-up. Detailed data were collected from all participants on reproductive outcomes and sexual behavior at each of three time points: baseline (T_0_), first follow-up (T_1_) which occurred between 1 year and 18 months after baseline, and second follow-up (T_2_) which occurred between 1 year and 18 months after the first follow-up data collection. The amount of time participants were followed up is controlled for in analyses by creating a variable of time since baseline data collection. The date of the baseline interview was subtracted from the final interview.

### Data analysis

Data analysis was carried out in Stata 10 (Stata Corp., College Station, TX, USA). Descriptive statistics were first calculated for all variables. Potential risk and protective factors and incident unwanted or unplanned pregnancies were explored. Two-way associations were determined between measures of gender power relations including relationship control and IPV variables, sexual risk behavior such as number of sexual partners, and demographic variables. For continuous variables the summary took the form of means with 95% confidence intervals (95% CIs), whereas for binary variables the summary took the form of percentages with 95% CIs. Variables were considered statistically significant if *p*<0.05.

To assess risk and protective factors for incident unwanted or unplanned pregnancies, we constructed multivariate polytomous regression models using svy: mlogit, which adjusts for clusters as latent variables within the model. Variables were considered for inclusion in the model if the significance in two-way analyses was less than 0.2 or if they were theoretically important.

Explanatory variables included age at baseline, having a pregnancy prior to baseline, education, time between interviews, study intervention arm, mutual main partners, contraceptive use, experience of IPV, and socioeconomic status. Elimination was used in the modeling to obtain the most parsimonious model. Variables that had a non-significant *p*-value were systematically eliminated with the least significant variable eliminated first. All IPV variables and relationship control were included in the original model and systematically eliminated, with the exception of experience of IPV by type of violence. All theoretically relevant interaction terms were tested and none were found to be statistically significant.

### Ethical considerations

Ethical clearance for the study was granted by the University of Pretoria ethics committee. Written consent was obtained for all participants recruited into the study.

## Results

Eight hundred and nineteen study participants provided data at baseline and at least one follow-up time point. These participants reported a total of 174 pregnancies over approximately 2 years of follow-up. Of these pregnancies, 10 (3.6%) were wanted at that time, and these respondents were dropped from subsequent analyses. Of the remaining 164 pregnancies, 53 (32.3%) were unplanned and 85 (67.7%) were unwanted. Pregnancy intention data were missing for 22 young women, and they were also dropped from analyses, for a total of 756 women reporting 136 pregnancies.

Table 1 shows the sociodemographic and select sexual and reproductive behaviors for three groups: women who reported no incident pregnancy, and those who had at least one unplanned or unwanted pregnancy at T1 or T2. Those women who reported an unwanted or unplanned pregnancy came from households with lower socioeconomic status. Young women who had incident unplanned or unwanted pregnancies were less likely to have had a pregnancy prior to baseline. They were also less likely to report hormonal contraceptive use at baseline and more likely to report using only condoms as contraceptives. They were more likely to believe that they were their boyfriend's main partner, and have a relationship of shorter duration.

**Table 1:** Sociodemographic and behavioral characteristics of teenage girls (15–18) by whether they reported no pregnancy or incident unplanned or unwanted over approximately 2 years of follow-up

Demographic variables	No incident pregnancy (*n*=620)			Incident unplanned pregnancies (*n*=51)			Incident unwanted pregnancies (*n*=85)			
			
	*n*	%	95% CI	*n*	%	95% CI	*n*	%	95% CI	*p*
Age at baseline										0.67
15 years	23	3.65	2.3–5.7	1	1.85	0.3–12.5	4	4.55	1.7–11.7	
16 years	148	23.49	20.4–26.8	12	22.22	12.0–37.4.5	17	19.32	13.1–27.5	
17 years	251	39.84	35.5–44.4	27	50.0	34.7–65.3	42	47.73	35.7–60.0	
18 years	208	33.02	28.9–37.4	14	25.22	15.0–40.9	25	28.41	17.7–42.3	
Boyfriend in past 12 months	609	96.67	95.1–97.8	52	96.3	86.1–99.1	87	98.9	92.2–99.8	0.43
Mutual main partners	458	72.70	68.4–76.6	46	85.19	71.8–92.9	71	80.7	68.1–89.1	0.11
10 or more years of education	276	43.81	36.7–51.2	21	38.89	26.4–53.0	35	40.23	27.4–54.6	0.69
Mean socioeconomic status score	629	0.11	−3.1–3.8	54	–0.47	−3.1–2.3	88	–0.14	−3.1–3.8	<0.01
Mean duration of main relationship	615	23 mo	0–148	53	21 mo	0–68	85	19 mo	0–74	0.04
Mean no of days since last sex	545	107 days	0–1170	51	114 days	1–2100	82	77 days	1–870	0.16
Pregnancy prior to baseline	83	14.72	11.6–18.6	7	13.46	6.3–26.6	3	3.61	1.2–10.5	0.02
Contraception										0.02
None	221	39.89	35.1–44.9	29	55.77	43.2–67.7	34	40.96	30.9–51.0	
Hormonal	245	43.44	38.5–48.6	14	26.92	15.9–41.8	27	32.53	23.2–43.0	
Condoms only	94	16.67	13.3–20.7	9	17.31	9.0–30.6	22	26.51	19.7–34.0	
Contraceptive knowledge										0.64
Low	262	41.72	38.1–45.5	26	48.15	33.2–63.5	39	42.47	34.9–54.0	
High	366	58.28	54.5–62.0	28	51.85	36.5–66.8	49	55.68	45.8–65.0	
Risky behavior										
Number of sexual partners at baseline										0.86
0	29	5.32	3.6–8.0	3	5.88	2.0–16.2	3	3.66	1.2–10.6	
1	340	62.39	58.0–66.6	30	58.82	42.9–73.1	52	63.41	52.8–72.9	
2 or more	176	32.29	28.5–36.4	18	35.29	22.1–51.3	27	32.93	23.2–44.3	

### Loss to follow-up

Table 2 shows that of the (*n*=922) women aged 15–18 years, 11.4% (*n*=103) were lost to follow-up. These women were older and were less likely to have completed 10 years of schooling than those who were successfully followed; those lost to follow-up were also more likely to have been sexually active at baseline. The mean age of the teenage girls retained in the cohort was 17.04 years (range 15–18 years). At baseline, 87.3% of participants had had sexual intercourse. By the end of the follow-up period, 93.6% of the young women had had sexual intercourse.

**Table 2:** Comparison of the study participants followed up and lost to follow-up

	Followed up (%) (*n*=819)	Lost to follow-up (%) (*n*=103)	*p*
Age (Mean years)	17.0	17.3	<0.01
Education:>grade 10	43.5%	30.1%	<0.01
Socioeconomic status (score)	0.05	0.03	0.90
Ever had a boyfriend	96.1	98.1	0.32
Ever had sex	87.3	94.2	0.05
Pregnancy prior to baseline	13.7	12.5	0.73
Mutual main partner	76.6	83.2	0.10
Duration of sexual activity (years)	2.24	2.24	0.99
Alcohol problem	3.5	6.8	0.14
Lifetime Number of sexual partners			0.64
≤1	67.5	71.9	
2–5	29.9	25.0	
>5	2.7	3.1	
Contraceptive use – ever	68.3	69.4	0.88
Age difference with partner (years)	3.11	2.77	0.15
Experience of IPV ever by type			
None	68.07	60.0	0.14
Physical abuse only	20.69	27.0	
Physical and/or sexual abuse	11.24	13.0	
Reproductive health knowledge score	23.0	22.4	0.14
Intervention: Stepping Stones	53.0	56.3	0.57

### Relationship between unwanted and unplanned pregnancies and gender-based violence and power and control in relationships

Table 3 shows the associations between experiences of gender-based violence and power and control in relationships and incident unwanted and unplanned pregnancies. Young women who had an incident unplanned and unwanted pregnancy were more likely to report experiences of physical abuse at baseline than those women who did not have an incident pregnancy. However, they were less likely to report experiences of sexual abuse. There was no association between power and control in relationship at baseline and subsequent incident unplanned or unwanted pregnancies. Having a non-consensual first sexual experience was also not associated with having a teenage pregnancy. Coerced sex under the age of 15 years was not associated with incident pregnancies. Child sexual abuse was associated with first pregnancies (some occurred prior to baseline), but not with subsequent pregnancies (44.1% vs. 31.9%; *p*=0.002) (data not shown in table).

**Table 3:** Associations between gender-based violence and control and incident pregnancies over approximately 2 years of follow-up

	No incident pregnancy (*n*=620)			Incident unplanned pregnancies (*n*=51)			Incident unwanted pregnancy (*n*=85)			
			
	*n*	%	95% CI	*n*	%	95% CI	*n*	%	95% CI	*p*
Lifetime experience of IPV by a boyfriend by type										0.06
None	437	69.81	65.9–73.5	38	70.37	58.2–80.2	57	64.77	54.9–73.5	
Physical abuse only	116	18.53	15.2–22.4	11	20.37	11.5–33.4	26	29.55	21.2–39.5	
Sexual abuse with/without physical	73	11.66	9.1–14.8	5	9.26	4.2–19.3	5	5.68	2.5–12.4	
Temporality of experience of IPV by a boyfriend										0.82
None	372	62.52	58.1–66.7	32	62.75	49.0–74.7	50	58.82	49.0–68.0	
Before the past 12 months only	37	6.22	4.4–8.7	3	5.88	1.9–16.8	4	4.71	1.8–12.0	
Within the past 12 months only	117	19.66	16.4–23.4	9	17.65	9.0–31.6	16	18.82	11.6–29.1	
Both before and within the past 12 months	69	11.60	9.2–14.6	7	13.73	6.7–26.1	15	17.65	11.7–25.8	
Experience of IPV by a boyfriend by frequency										0.16
None	407	64.60	60.5–68.5	35	64.81	52.3–75.6	53	60.23	50.4–69.3	
Once only	108	17.14	14.2–20.6	4	7.41	2.7–18.9	18	20.45	13.3–30.1	
More than once	115	18.25	15.3–21.7	15	27.78	17.7–40.8	17	19.32	13.3–30.1	
Relationship control scale										0.90
Low control (more equal)	189	29.0	25.4–35.0	15	27.78	17.2–41.5	29	32.95	22.8–45.0	
Medium control	315	50.0	45.7–54.4	26	48.15	33.7–62.9	44	50.0	38.7–61.3	
High control (less equality)	126	20.0	16.6–24.0	13	24.07	12.7–40.8	15	17.05	16.7–23.7	
Forced first sex (*p*=0.21)	85	13.49	10.9–16.7	5	9.26	4.3–18.9	8	9.09	4.1–18.9	0.94
Coerced sex before 15 (*p*=0.61)	20	3.67	2.4–5.6	3	5.88	2.0–16.3	4	4.88	2.0–11.2	0.64

### Multivariable analysis results

Table 4 shows the results from the polytomous regression on unwanted and unplanned pregnancies. Hormonal contraception was significantly protective against unplanned pregnancies; however, it showed no impact on unwanted pregnancies. Using condoms as contraceptives was not associated with unplanned pregnancies. Physical abuse was a risk factor for unwanted pregnancies, but not unplanned pregnancies. Lower socioeconomic status was a risk factor for both unplanned and unwanted pregnancies. Believing that the teenage girl and her boyfriend were mutual main partners doubled the odds of reporting both an unplanned and unwanted pregnancy. Having a pregnancy prior to baseline was protective against an unwanted pregnancy; however, this was not the case for unplanned pregnancies.

**Table 4:** Polytomous regression model of risk factors for incident unplanned and unwanted teenage pregnancies, adjusted for time between interviews and treatment arm

	OR	95% Confidence Interval		*p*
Incident unplanned pregnancies				
Pregnancy prior to baseline	1.01	0.39	2.64	0.98
Age	0.90	0.60	1.35	0.61
Relationship duration	1.00	0.98	1.01	0.58
Contraception – none	Ref.			
Hormonal	0.40	0.21	0.79	<0.01
Condoms only	0.86	0.38	1.99	0.73
Mutual main partners	2.58	1.07	6.25	0.03
Socioeconomic status	0.69	0.58	0.83	<0.01
Exposure to intervention	0.96	0.50	1.85	0.89
IPV – none	Ref.			
Physical abuse only	1.06	0.51	2.21	0.88
Sexual abuse with/without physical abuse	0.74	0.30	1.81	0.51
Incident unwanted pregnancies				
Pregnancy prior to baseline	0.25	0.07	0.80	0.02
Age	1.09	0.82	1.46	0.54
Relationship duration	0.98	0.97	1.00	0.06
Contraception – none	Ref.			
Hormonal	0.74	0.43	1.30	0.29
Condoms only	1.81	0.99	3.30	0.05
Mutual main partners	2.21	1.13	4.29	0.02
Socioeconomic status	0.78	0.64	0.96	0.02
Exposure to intervention	1.43	0.85	2.41	0.18
IPV – none	Ref.			
Physical abuse only	1.69	1.05	2.72	0.03
Sexual abuse with or without physical abuse	0.55	0.24	1.27	0.16

## Discussion

The study found that lower socioeconomic status and a teenage girl believing that she was her boyfriend's main partner were risk factors for both incident unwanted and unplanned pregnancies. Having had a previous pregnancy and using hormonal contraceptives were protective against unplanned but not unwanted pregnancies. Women who had experienced physical abuse were more likely to have an unwanted pregnancy.

Unlike other studies, contraceptive knowledge and educational attainment were not associated with incident pregnancies over the 2 years of follow-up (12, 13). Our study suggests that the relationship dynamics and access to health services may play a greater role in teenage pregnancies than knowledge and education, although it may also be the case that because participants were recruited from schools, their educational attainment was too homogenous to observe an effect.

This study explored multiple potential connections between the experience of violence and abuse and teenage pregnancy. Unlike some previous studies, the results of the current study do not support a link between forced first sex and teenage pregnancies (15, 21); however, child sexual abuse was associated with first pregnancies (although not with second or third pregnancies). Our findings thus support a link between experience of early trauma and early pregnancy, but suggest that cumulative trauma is more important than a single sentinel event. Similarly, although we did not observe an association between power and control in intimate relationships and incident pregnancies, we did show that young women who experienced physical abuse were more likely to report a new unwanted pregnancy. A parallel ethnographic study conducted among young women from the study cohort show that women reported feeling little-to-no ability to control their sexual or reproductive lives (30). Although the findings on violence and unwanted teenage pregnancy reported here reflect multiple nuanced links at the level of individual analysis, it is likely that the pervasive context of women's disempowerment, created in part through the common occurrence of violence, also contributes to unwanted pregnancy.

Lower socioeconomic status was a risk factor for both unplanned and unwanted pregnancies. Teenage girls who come from poorer families may perceive pregnancy as a way to ensure greater security for their future, if the paternity of the child is established and the father of the child's family provides economic support (31, 32). By contrast, teenage girls who come from families who are better off may have a greater fear of becoming pregnant and disappointing their families, negatively having an impact on their educational and social status (31). However, not all teenage pregnancies are stigmatized; there are families which embrace teenage pregnancies and view it as a rite of passage to womanhood (33).

Young women's beliefs about the nature of their relationship increased the likelihood of having an incident pregnancy over the follow-up period. In particular, teenage girls who believed that they and their boyfriends were mutual main partners were more likely to report both unwanted and unplanned pregnancies. The perceived stability of the relationship could lead teenage girls to feel less concerned about the possibility of becoming pregnant. This may lead to inconsistent contraceptive use. In addition, the association with physical abuse from a male partner, combined with believing themselves to be in a committed relationship could result in young women acquiescing to pressure to take risks and not use contraceptives (especially condoms) consistently (32).

Use of hormonal contraceptives was protective against unplanned pregnancies, whereas using only condoms was a risk factor for an unwanted pregnancy. This could be because of condom breakage or lack of commitment to consistent and correct use (34). We also found that having a prior pregnancy was protective against a future unwanted pregnancy. This is additional evidence of a fertility trend in South Africa reported by Garenne and his colleagues (35) who found that women who give birth as teenagers wait several years before having another child. Having a pregnancy also enables and encourages young women to interact with health care services. In South Africa, more than 90% of women attend antenatal care services at least once, and most women deliver in hospitals or maternal and obstetric units. This provides an opportunity for women to access contraceptive services after delivery.

There is an unmet contraceptive need among the young women in the Eastern Cape, evident by the high percentage of teenage girls having unplanned and unwanted pregnancies. Although these services are provided free of charge at primary health care clinics in South Africa, there are problems with access, especially for young women. Health care providers are often judgmental about teenagers seeking contraceptive services (31, 36). In addition, we found that the mean time since last sex was 3 months, indicating that many of the young women are having sex infrequently. This may affect the consistency of contraceptive use, especially the commonly used injectable contraceptives (34). The two injectables used in primary health care clinics are depot medroxyprogesterone acetate (DMPA) and norethisterone enantate (NET-EN). Both are progestin-only injectables with DMPA providing 12 weeks’ protection and NET-EN 8 weeks’ protection, requiring repeat visits to clinics (34). Because many of the young women in the study were having sexual intercourse less frequently, they may not have attended the clinic for their follow-up visit and the next dose of the injectable contraceptive. This finding suggests the need for a wider range of contraceptive methods and wider promotion of emergency contraceptives.

Our study has several limitations. Pregnancies were self-reported and it is possible that women did not disclose pregnancies that resulted in a termination. This may have resulted in under-reporting of pregnancies. Subsequent longitudinal studies could explicitly measure induced abortions and miscarriages. Participants were volunteers, and this may limit the generalizability of the findings. Teenage girls who were lost to follow- up were older, with lower educational attainment, and the effect of this differential loss on the outcomes investigated in our study is unknown. However, the key strength of the study is the longitudinal design with incident pregnancies occurring after the risk and protective factors under study. In addition, the study differentiates between the risk and protective factors for unwanted and unplanned pregnancies among adolescent women. This allows for a more nuanced response.

## Conclusion

Although not all of the measures of gender inequity were associated with unplanned and unwanted pregnancies in this study, there is evidence that inequitable gender power relations and low socioeconomic status do increase risk. Interventions to prevent teenage pregnancies need to be tailored by socioeconomic status because some teenagers may see having a pregnancy as a way to have a more secure future. Interventions that engage with teenagers’ relationship dynamics are essential for effective prevention of unwanted and unplanned pregnancies. There is also an unmet contraceptive need among the young women in the Eastern Cape.
